# Digital Tools to Support Post‐Secondary Student Mental Health and Wellbeing

**DOI:** 10.1111/eip.70094

**Published:** 2025-10-07

**Authors:** Haley M. LaMonica, Ian B. Hickie, William Capon, Maya Ahia, Lexi Ewing, Wendy Lee, Frank Iorfino, Yun J. C. Song, Sarah McKenna, Kristin Cleverley

**Affiliations:** ^1^ Youth Mental Health and Technology Team, Brain and Mind Centre The University of Sydney Sydney Australia; ^2^ The Graduate Department of Psychological Clinical Science University of Toronto Toronto Canada; ^3^ Centre for Addiction and Mental Health Toronto Canada; ^4^ Sydney School of Education and Social Work The University of Sydney Sydney Australia; ^5^ Bloomberg Faculty of Nursing University of Toronto Toronto Canada

**Keywords:** accessibility, co‐design, digital mental health technology, emerging adulthood, mental health, post‐secondary education, public health

## 1 Primary Argument

Digital technologies have acted as a revolutionising force across diverse industries, including addressing health system and accessibility challenges (Botelho 2021). The scalability and cost‐effectiveness of digital technologies are essential to meet the growing demand for mental health care (McGorry et al. 2024), enabling assessment, intervention delivery, and, importantly, routine outcome monitoring to ongoingly inform personalized recommendations about self‐care, clinical, and psychosocial supports and interventions to promote better outcomes. Given the ubiquity of smartphones and internet use, particularly amongst young people, we argue that digital technologies are the only viable option to support both the mental health and academic success of post‐secondary students, with data collection capabilities serving to inform the delivery of institutional services and supports that fit the needs of the student body and enable the coordination of care with traditional health systems.

## 2 The Mental Health of Post‐Secondary Students

Global trends indicate mental health has worsened amongst emerging adults in recent decades (McGorry et al. 2024), contributing to a reduced life expectancy by approximately 15 years and a major lifelong burden that impacts individuals, their families, and communities globally (Jones 2013). Emerging adulthood often overlaps with the transition into post‐secondary education (i.e., college or university), an already challenging life stage that can exacerbate vulnerability to mental health problems (Lipson et al. 2022; Solmi et al. 2022). As a result, the prevalence and complexity of mental health‐related challenges amongst post‐secondary students have become an increasing concern on college and university campuses worldwide. Notably, almost one‐third of post‐secondary students meet diagnostic criteria for a mental disorder (Kieling et al. 2024), relative to global prevalence rates of 13.96% and 13.63% for young people aged 15–19 years and 20–24 years respectively (Kieling et al. 2024).

Emerging adults in post‐secondary education have unique needs and experiences that warrant greater consideration within mental health policy and research (Byrom et al. 2025). Perhaps most obviously, post‐secondary education is associated with heightened academic demands, standards, and expectations often associated with anxiety and fear of failure (Cage et al. 2021; Lisnyj et al. 2021; Wilbraham et al. 2024), with outcomes touted as being directly linked to future employment opportunities (Larcombe et al. 2022). Many students are also confronted with significant financial burdens (Larcombe et al. 2022), new living circumstances outside of home (Worsley et al. 2021), social isolation without easy access to family and friends (Worsley et al. 2021; Diehl et al. 2018), and a significantly increased need to be self‐reliant and independent (Lisnyj et al. 2021; Wilbraham et al. 2024). This is particularly true for students with mental health‐related disabilities who may face challenges navigating post‐secondary environments that are not innately accessible (Tan et al. 2023). Further, some students appear to be at an increased risk for functional impacts of mental illness (Iorfino et al. 2022). For example, in Australia, First Nations students and those from lower socioeconomic households and regional and rural communities are more likely to disengage from coursework due to health and stress‐related reasons relative to high‐socioeconomic, metro, and non‐Aboriginal and Torres Strait Islander students (Edwards 2015), a finding also evident in other parts of the world such as Canada (Shankar et al. 2013).

Post‐secondary student mental health is a priority for post‐secondary institutions (Cecil 2021; Clark and Morgan 2021; Melidona et al. 2021), prompting the development of national frameworks (Baik et al. 2017; Baik et al. 2016; Hughes and Spanner 2019; Mental Health Commission of Canada Canadian Standards Association Group 2020). Despite these standards, there remains a gap in the evidence for what supports and interventions are most effective, for whom, and in what contexts. The digital health sector is a developing market, coinciding with increasing demand for evidence‐based solutions for mental health (Ridout et al. 2024); however, its potential remains underexplored for the post‐secondary school context.

## 3 Digital Technologies

Digital mental health refers to the use of digital technologies in mental health care, including for “mental health and wellbeing promotion and prevention, wellbeing maintenance/self‐care, early intervention, or for treating specific mental illnesses” (Bond et al. 2023). Digital mental health technologies include mobile health applications (e.g., mindfulness applications, digital diaries), wearable devices (e.g., sleep trackers), telehealth videoconferencing platforms, remote monitoring devices, web‐based platforms (e.g., Innowell [Iorfino et al. 2019]), and AI chatbots (Bond et al. 2023). These technologies have demonstrated effectiveness for young adults in detecting both emerging and fully manifest mental disorders (McDonald et al. 2019), monitoring clinical and functional outcomes to predict personal‐level change (Iorfino et al. 2019; LaMonica et al. 2022; Oudin et al. 2023), providing services via text message, web‐based platforms, mobile applications, and virtual reality (Wies et al. 2021); and coordinating care across a health system (Iorfino et al. 2021). Additionally, digital technologies continue to play a critical role in decreasing access barriers for those with disabilities and diverse accessibility needs (Botelho 2021).

The promise of technology to transform mental health care is being applied in post‐secondary settings, where most students have access to smartphones and report preferences for flexible, low‐barrier methods of seeking care (Lungu and Sun 2016; Bautista and Schueller 2023). Reviews suggest digital mental health interventions can benefit symptoms of depression and anxiety in student populations, which are amongst the most prevalent mental health symptoms reported by post‐secondary students worldwide (Kieling et al. 2024; Tan et al. 2023; Lattie et al. 2019; Alagarajah et al. 2024). These remote interventions can support the delivery of personalised care that allows students to flexibly seek care at times best suited to their needs, particularly around busy timetables (Bautista and Schueller 2023; Cohen et al. 2022). However, despite growing availability, uptake and sustained adoption remain an issue (Kern et al. 2018; Melcher et al. 2022). Simply making digital mental health technologies available to students is not an effective strategy for sustaining engagement (Bautista and Schueller 2023) or achieving desired outcomes (Garrido et al. 2019).

## 4 Misalignment of Treatment Needs and Digital Tools

There are two interacting reasons why poor uptake, adoption, and sustained engagement with digital technologies is present in post‐secondary settings. First, we do not fully understand what students' treatment needs are from digital mental health technologies, which may mean that interventions lack specificity for this group, leading to poor uptake and adoption. Second, we do not have adequate evidence on what outcomes these tools can deliver in institutional settings, given the unique needs of post‐secondary students. That is, blind spots on both the intervention side (not knowing if X works for Y) and the target side (not knowing whether students even have Y) exist. Treatment needs in this context do not refer to things like safety (Lattie et al. 2020), accessibility, and usability (Lattie et al. 2019), which are established factors necessary for effective digital technologies in this setting, but rather the specific mental health challenges (symptom profiles, functional impairment, loneliness) that could be appropriately responded to with technology. A recent systematic review and meta‐analysis found that digital mental health interventions are effective for post‐secondary students experiencing anxiety or depression; however, there was considerable heterogeneity in the results that could stem from a mismatch between student needs and intervention type (i.e., with or without human support) and psychological treatment (e.g., cognitive‐behavioural therapy, multicomponent) (Madrid‐Cagigal et al. 2025). The uncertainty about what works and what is needed has led to a standstill, forcing post‐secondary institutions into a state of overwhelm with the abundance of digital mental health technologies available. Rising pressure from commercial bodies to adopt and implement apps at campus scale is burdening post‐secondary institutions to make decisions while being under‐equipped to act informatively. Addressing these gaps requires intentionally centering students in both research and development to design more effective, engaging, and responsive interventions that go beyond traditional models of care. This will inevitably involve helping students navigate between traditional health care for those with more complex needs and specific social and educational supports as required.

Participatory co‐design not only improves usability and relevance but also helps to identify the real‐world needs, preferences, and lived experiences that should guide digital intervention design and evaluation (Collins et al. 2018; Malloy et al. 2023; Orlowski et al. 2016). Without authentic student co‐design, institutions may implement tools that are misaligned with the student experience and needs, and therefore unlikely to benefit them meaningfully or meet institutional priorities. When students are meaningfully involved in the development process, from ideation through testing, interventions are more likely to be adopted and maintained over time (Malloy et al. 2023). Additionally, a blended care model that combines digital tools with face‐to‐face human support (Bautista and Schueller 2023; Igoe 2024) may optimise outcomes and satisfaction. Indeed, human support has been shown to be critical to achieving desired outcomes via digital mental health technologies (Bautista and Schueller 2023; Garrido et al. 2019; Igoe 2024; Lehtimaki et al. 2021). This suggests that simply shifting students toward digital technologies as a substitute for face‐to‐face care is unfavourable for engagement. Furthermore, conceptualising digital mental health technologies not just as tools but as social infrastructures that can either inhibit or promote accessibility and inclusion helps us understand how to engage in meaningful co‐design with students with diverse mental health‐related accessibility needs (van Toorn 2024).

## 5 Data Silos and Missed Opportunities

To guide meaningful investment and advancement in the digital mental health space, post‐secondary institutions need to invest and engage in evaluation research that extends beyond uptake and engagement to include long‐term clinical and functional (i.e., academic performance) outcomes (Abelson et al. 2024; Wiljer et al. 2020), led in partnership with students. Mental health, mental health care accessibility, and academic performance are strongly intertwined. Delays in receiving timely, accessible mental health care can exacerbate existing mental health symptoms and may alienate students from engaging in help‐seeking behaviours more generally in the future. This cycle often results in missed opportunities for primary prevention and early mental health intervention, leading to students falling further through the cracks with worsening health and academic outcomes (Moghimi et al. 2023). Both internalising (e.g., depression, anxiety, sleep disturbance) and externalising (e.g., inattentiveness, impulsivity) mental health problems have been shown to be associated with significant declines in academic performance (i.e., lower GPAs) amongst post‐secondary students (Bruffaerts et al. 2018). However, the potential causality of these relationships requires further exploration (Bruffaerts et al. 2018) as academic performance difficulties appear to be primarily driven by the functional impacts of mental illness (Holmes and Silvestri 2016). For example, post‐secondary students with an anxiety disorder are more likely to experience executive dysfunction and memory problems, whereas those with a mood disorder report greater difficulties with attention (Holmes and Silvestri 2016). While there is a growing evidence base supporting the effectiveness of digital mental health technologies, particularly as an adjunct to clinical care (Fuhrmann et al. 2024) or when used with human support (Garrido et al. 2019; Lehtimaki et al. 2021), these tools rarely report effects on academic performance (Bolinski et al. 2020), which leaves a gap in understanding the impact of these tools in this setting.

Unfortunately, this gap is mirrored by most institutional evaluation practises. For example, 10 directors of post‐secondary university counselling or health and wellness centres in Canada recommended four categories of metrics for routine monitoring, including programme usage, student characteristics and treatment outcomes, perceived value, and staff experience (Lattie et al. 2019). However, academic outcomes and institutional costs were notably absent. While many post‐secondary institutions already collect rich datasets on academic performance, service utilization, disengagement rates, and post‐graduation outcomes, these data are often siloed across departments and governed by varying privacy regulations (e.g., Personal Health Information Protection Acts), making data integration challenging (Baharom et al. 2025). Consequently, the relationship between students' mental health and their academic and institutional outcomes remains under‐investigated. Co‐designed approaches can play a pivotal role in addressing this gap, not only in aligning interventions with student needs, but in ensuring evaluation strategies include outcomes that reflect both academic and personal wellbeing.

Without integrated data and collaborative governance, institutions are missing a major opportunity to understand the broader return on investment in mental health services, such as reduced tuition loss and improved long‐term alumni donations/engagement, which have been demonstrated to have positive effects where support is effectively implemented (Ashwood et al. 2016). Further, connecting these data streams would highlight the students most at risk of poor mental health outcomes and disengagement, such as students with diverse mental health‐related accessibility needs, which would support the validation of (digital) interventions against meaningful long‐term outcomes. Indeed, there have been global calls for enhanced data collection on post‐secondary student mental health, including longitudinal and mixed methods approaches, to inform institutional‐ and system‐level policies and programmes (Mental Health Commission of Canada Canadian Standards Association Group 2020; Browne et al. 2017). This would both enable personalised responses for individuals as well as inform local and regional health system responses. Thus, formalising an integrated data strategy, informed through co‐design, as part of institutional work, health, and safety policies would support accountable decision‐making that improves mental health service design and delivery and ensures investments are aligned with academic success and productivity.

## 6 Call to Action Toward Coordinated Digital Mental Health

If we are to reflect on the broader lessons of the digital mental health boom in the youth mental health field, we can clearly identify the role digital technologies play in delivering effective, accessible mental health care and collecting comprehensive data (Hickie et al. 2025; Capon et al. 2023). Yet even when engagement with technologies is high, their efficacy on long‐term post‐secondary students' clinical and academic outcomes is unclear. To move forward, coordinated and evidence‐informed strategies are needed to align digital tool development and evaluation with students' needs and institutional priorities. This includes focusing on co‐design practises that embed students across the development, implementation, and evaluation processes to ensure that the digital interventions respond to real needs and are assessed against outcomes that matter to students. Additionally, institutions must adopt integrated data strategies that break down data silos, allowing for more holistic assessments of impact. Together, these approaches will support a more accountable, student‐centred digital mental health ecosystem that drives meaningful improvement in student wellbeing and academic outcomes that are aligned with institutional needs and priorities.

## Ethics Statement

The authors have nothing to report.

## Conflicts of Interest

Ian B. Hickie is the Co‐Director, Health and Policy at the Brain and Mind Centre (BMC), University of Sydney. The BMC operates an early‐intervention youth services at Camperdown under contract to headspace. He is the Chief Scientific Advisor to, and a 3.2% equity shareholder in, InnoWell Pty Ltd. which aims to transform mental health services through the use of innovative technologies. The other authors declare no conflicts of interest.

## Data Availability

Data sharing not applicable to this article as no datasets were generated or analysed during the current study.
